# Contractility of temporal inverted internal limiting membrane flap after vitrectomy for macular hole

**DOI:** 10.1038/s41598-021-99509-0

**Published:** 2021-10-08

**Authors:** Akira Hirata, Keiko Mine, Ken Hayashi

**Affiliations:** 1 Hayashi Eye Hospital, 4-23-35, Hakataekimae, Fukuoka, Fukuoka 812-0011 Japan; 2 Department of Anatomy, Kurume University School of Medicine, 67 Asahi-machi, Kurume, Fukuoka 830-0011 Japan

**Keywords:** Adverse effects, Retinal diseases, Three-dimensional imaging

## Abstract

We investigated the postoperative visual outcomes and morphological changes of the internal limiting membrane (ILM) flap, in patients who underwent the temporal inverted ILM flap technique for macular hole (MH). Between August 2018 and February 2020, 22 eyes of 22 patients with idiopathic or myopic MH who underwent vitrectomy with ILM flap were included in this study and followed-up for more than 6 months. Postoperative MH status, comparison of best-corrected visual acuity (BCVA) before and 6 months after surgery, changes in the ILM flap area at 1 and 6 months postoperatively, and the factors related to changes in ILM flap size, were analyzed. MH closure was achieved in all of the patients. The BCVA at 6 months postoperatively (0.18 ± 0.15) was significantly better than the preoperative BCVA of 0.63 ± 0.37 (*P* < 0.001, paired *t* test). The area of the ILM flap decreased significantly from 3.25 ± 1.27 mm^2^ at 1 month to 3.13 ± 1.23 mm^2^ at 6 months (*P* = 0.024, Wilcoxon signed-rank test). Two eyes showed an ILM flap contraction of more than 20%, and one eye required reoperation due to an increase in metamorphopsia and decreased visual acuity. Among age, sex, ILM flap area at 1 month, preoperative BCVA, and axial length, ILM flap contraction was correlated with patient age and ILM flap area. Although vitrectomy with the inverted ILM flap technique confers a good visual outcome, the ILM flap may contract in younger patients.

## Introduction

Since the usefulness of vitrectomy for macular holes (MHs) was reported by Kelly and Wendel, surgical treatment for MH has become possible^1^. Furthermore, with the introduction of internal limiting membrane (ILM) peeling, both postoperative anatomical MH closure and improvement of visual function have been achieved with good and stable results^2^. However, in some cases, such as large MH, long-term MH, and myopic MH, treatment was difficult^3^. Michalewska et al. reported that the ILM flap technique can close refractory MH and continuously improve the foveal architecture^4^. Subsequent papers also reported that the ILM flap technique is superior in closing refractory MH^5–9^. The ILM flap technique has been modified from the original technique by Michalewska et al., which might more appropriately be called "ILM insertion," to various other techniques, including the creation of a single ILM flap covering the MH^10,11^.

On the other hand, placing the ILM flap over the macula may create a scaffold for cell proliferation, which may lead not only to MH closure but also to epiretinal membrane-like proliferative tissue formation. In our previous report, we described a case of MH with macular pucker due to contraction of a single sheet of the ILM flap created from the temporal side after vitrectomy with silicone oil injection^12^. However, there are no other reports on the changes that occur in the ILM flap after vitrectomy with gas tamponade.

With the advancement of optical coherence tomography (OCT), it has become possible to capture changes in the fine structure of the retina, and to quantitatively observe changes in the vitreous-retinal interface, including the shape of the ILM flap. In this study, we investigated changes in postoperative MH status and visual acuity after vitrectomy for MH, using the inverted ILM flap technique. Furthermore, changes in the ILM flap were compared between 1 month and 6 months after vitrectomy, and the clinical factors affecting the changes in the ILM flap were examined.

## Results

This study included 22 eyes of 22 patients who underwent vitreous surgery for MH. The MHs were idiopathic in 13 eyes and myopic in nine eyes. The clinical characteristics of the patients are summarized in Table 1. Compared to the myopic MH group, the idiopathic MH group was significantly older, had a larger MH size, and had poor preoperative BCVA.

**Table 1:** Patient preoperative clinical characteristics.

	Total (n = 22)	Idiopathic MH (n = 13)	Myopic MH (n = 9)	P-value
Patient age (years)	62.9 ± 11.0	68.3 ± 5.9	55.0 ± 12.1	0.003
Sex (male:female)	14:8	9:4	5:4	0.660
MH size (µm)	411.1 ± 178.3	492.5 ± 172.1	293.4 ± 112.9	0.007
Preoperative BCVA	0.63 ± 0.37	0.81 ± 0.37	0.37 ± 0.11	0.001
Axial length (mm)	25.45 ± 2.18	24.03 ± 1.15	27.55 ± 1.31	< 0.001

The values are presented as the mean ± standard deviation.

When comparing the clinical characteristics of patients with idiopathic MH and myopic MH, an unpaired *t* test was used for patient age, MH size, preoperative BCVA, and axial length, and Fisher's exact test was used for sex.

*MH* macular hole, *BCVA* best-corrected visual acuity.

**Figure 1:**
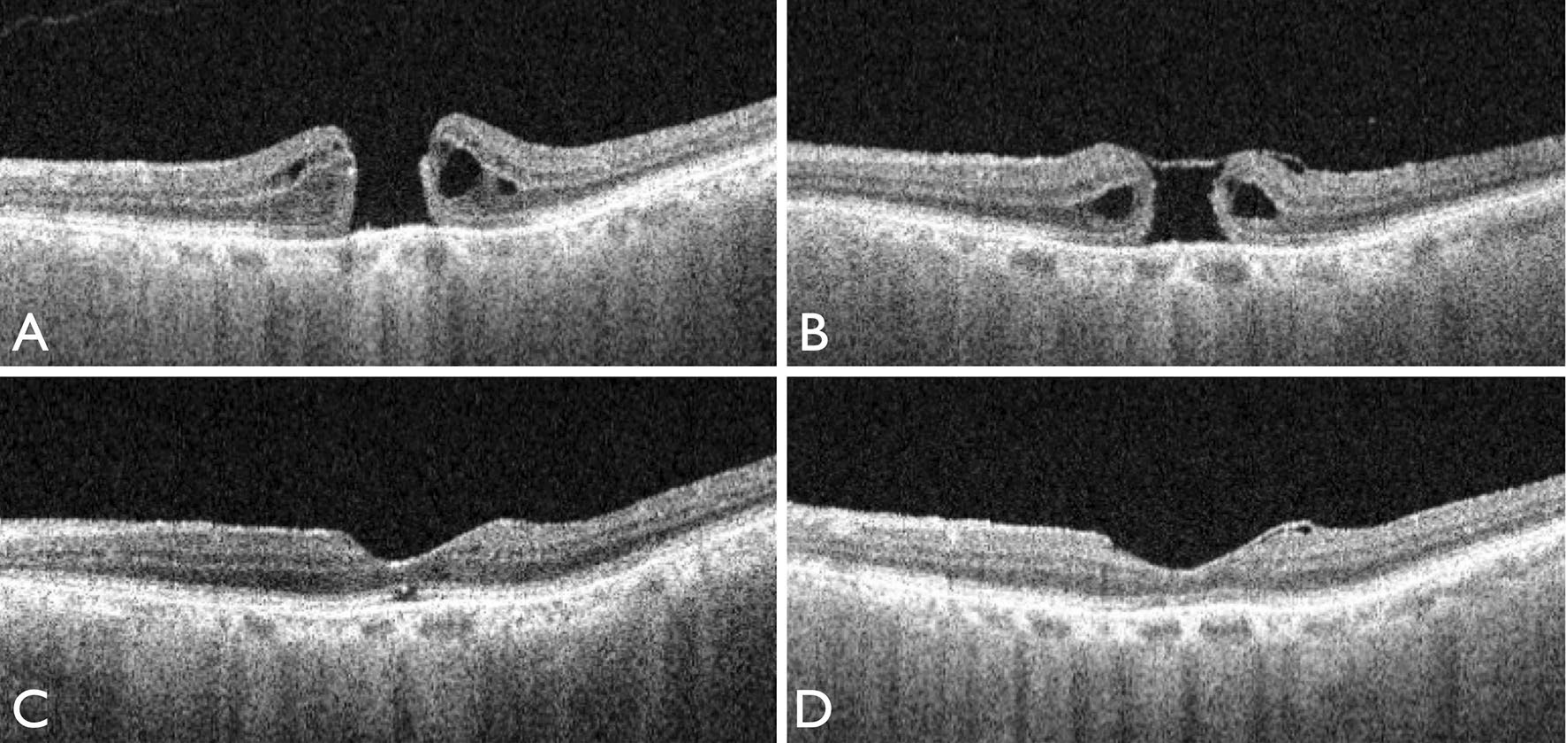
OCT images of a patient before and after macular hole (MH) surgery. (**A**) Preoperative OCT image. (**B**) OCT image obtained 1 week after vitrectomy. The hole remained open after the air in the vitreous cavity had disappeared. However, the ILM flap covered the MH. (**C**) OCT image 1 month after surgery, the MH was closed. However, the ellipsoid zone was disrupted. (**D**) OCT image at 6 months postoperatively; the continuity of the ellipsoid zone was restored.

MH closure was observed in 16 of the 22 eyes (73%) at 1 week after vitrectomy. In the remaining 6 eyes, although the MH was still open, the ILM flap covered the MH. The MH was closed in 21 of the 22 eyes (95%) at 1 month and in 100% of the 22 eyes at 1.5 months. Of the 22 eyes, restoration of continuity of the external limiting membrane (ELM) was seen in 4 eyes (18%) at 1 week, 15 eyes (68%) at 1 month, and 20 eyes (91%) at 6 months. Of the 22 eyes, recovery of the ellipsoid zone continuity was seen in 0% of eyes at 1 week, 11 eyes (50%) at 1 month, and 16 eyes (73%) at 6 months postoperatively (Fig. 1).

**Figure 2:**
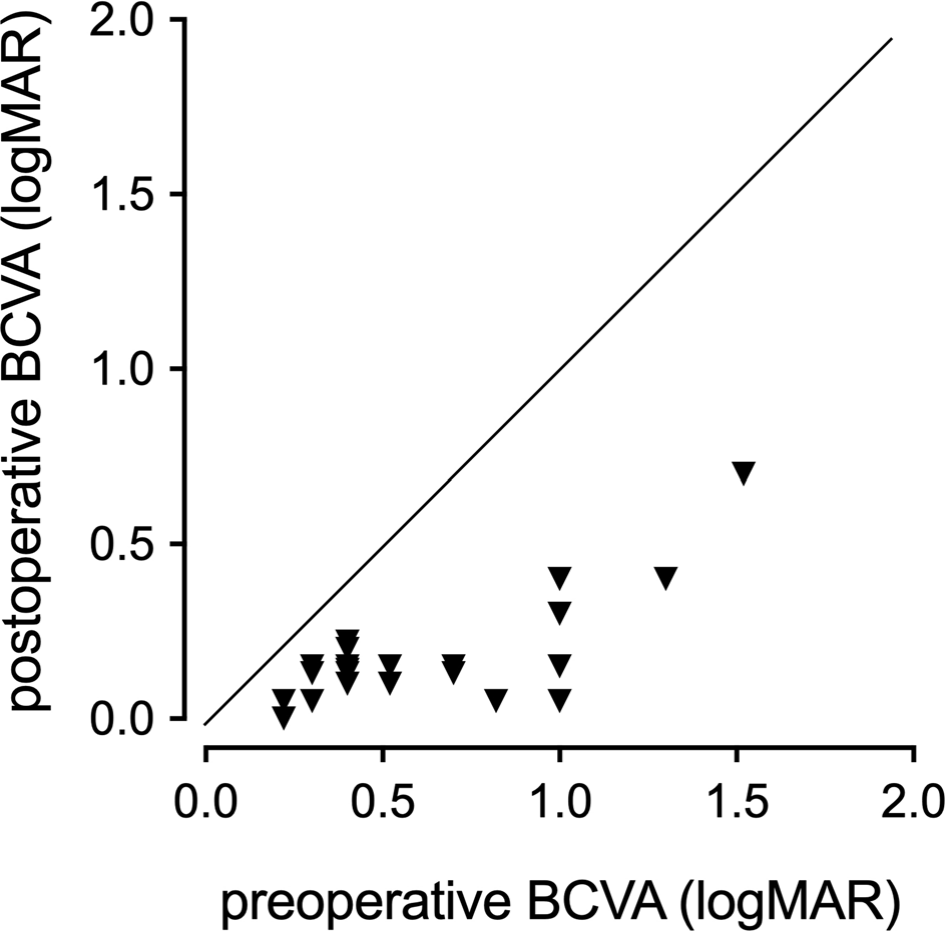
Best-corrected visual acuity (BCVA) before and 6 months after surgery. The preoperative BCVA was 0.63 ± 0.37 and the BCVA at 6 months after surgery was 0.18 ± 0.16. The postoperative BCVA was significantly improved compared to the preoperative BCVA (*P* < 0.001, paired *t* test).

The mean BCVA (log MAR) at 6 months postoperatively was 0.18 ± 0.16, which was significantly better than the preoperative BCVA of 0.63 ± 0.37 (*P* < 0.001, paired *t* test; Fig. 2). Correlation analyses revealed that postoperative BCVA after 6 months was significantly correlated with MH size (*r* = 0.603, *P* = 0.003), preoperative BCVA (*r* = 0.727, *P* < 0.001), and the size of the ILM flap at 1 month (*r* = 0.468, *P* = 0.028). Multivariate linear regression analyses with stepwise regression revealed that preoperative BCVA was a significant factor associated with postoperative BCVA (*β* = 0.727, *P* < 0.001).

**Figure 3:**
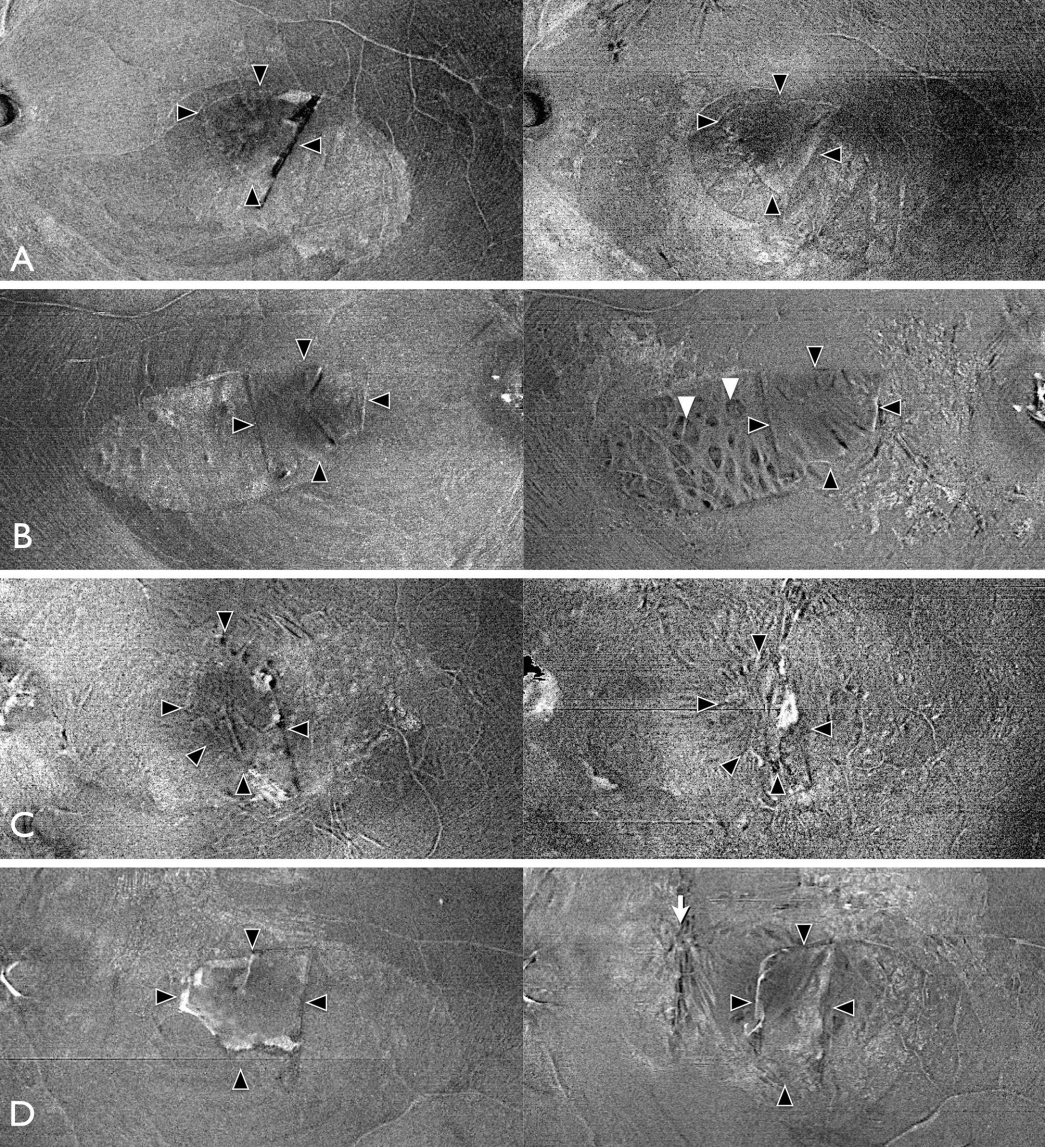
En face images of the internal limiting membrane (ILM) flaps. The left panel shows the findings at 1 month postoperatively, and the right panel shows the findings at 6 months, postoperatively. Cases with little change in the ILM flap area between 1 month and 6 months postoperatively (**A**,**B**) and cases with more than 5% contraction of the ILM flap at 6 months postoperatively (**C**,**D**). (**A**) A 72-year-old patient with idiopathic MH. The ILM flap over the macular hole (MH) (surrounded by arrowheads) remains exactly the same shape 6 months after surgery. The ILM flap area was 2.192 mm^2^ at 1 month postoperatively and 2.134 mm^2^ 6 months postoperatively. (**B**) A 73-year-old patient with idiopathic M. Although the ILM flap (arrowheads) retained the same shape at 6 months postoperatively, the ILM flap showed increased wrinkling. Inner retinal dimpling (white arrowheads) was observed in the area where the ILM was peeled to create the flap. The ILM flap area was 3232 mm^2^ at 1 month postoperatively and 3262 mm^2^ 6 months postoperatively. (**C**) A 72-year-old patient with idiopathic MH. The ILM flap (arrowheads) contracted centripetally at 6 months postoperatively, and wrinkles were observed on the retinal surface as the ILM flap contracted. The ILM flap area was 3.543 mm^2^ at 1 month postoperatively and 3.318 mm^2^ 6 months postoperatively. (**D**) A 45-year-old patient with myopic MH. The ILM flap (arrowheads) contracted and curled at the nasal side of the flap at 6 months postoperatively; ERM formation was also observed at the edge of the ILM peeled area (arrow). The ILM flap area was 3.601 mm^2^ at 1 month postoperatively and 2.776 mm^2^ 6 months postoperatively.

**Figure 4:**
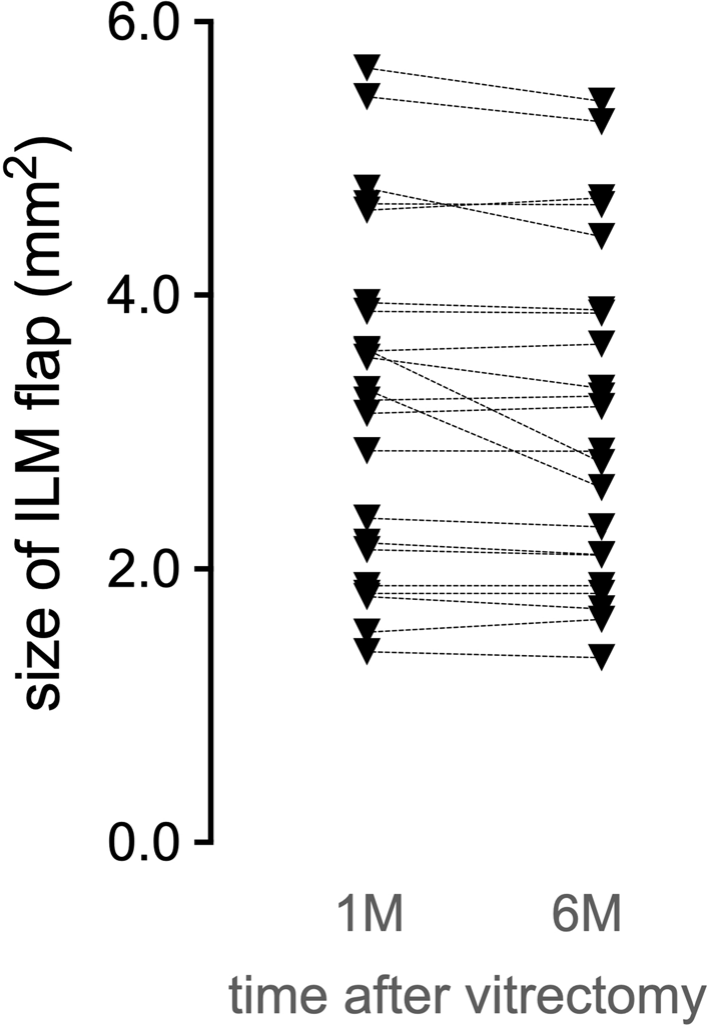
Area of the internal limiting membrane (ILM) flap 1 month and 6 months after surgery. The area of the ILM flap was significantly reduced from 3.25 ± 1.27 mm^2^ from 1 month to 3.13 ± 1.23 mm^2^ to 6 months (*P* = 0.024, Wilcoxon signed rank test). Five eyes (23%) showed ≥ 5% ILM flap contraction, of which two eyes had ≥ 20% contraction of ILM flaps.

**Figure 5:**
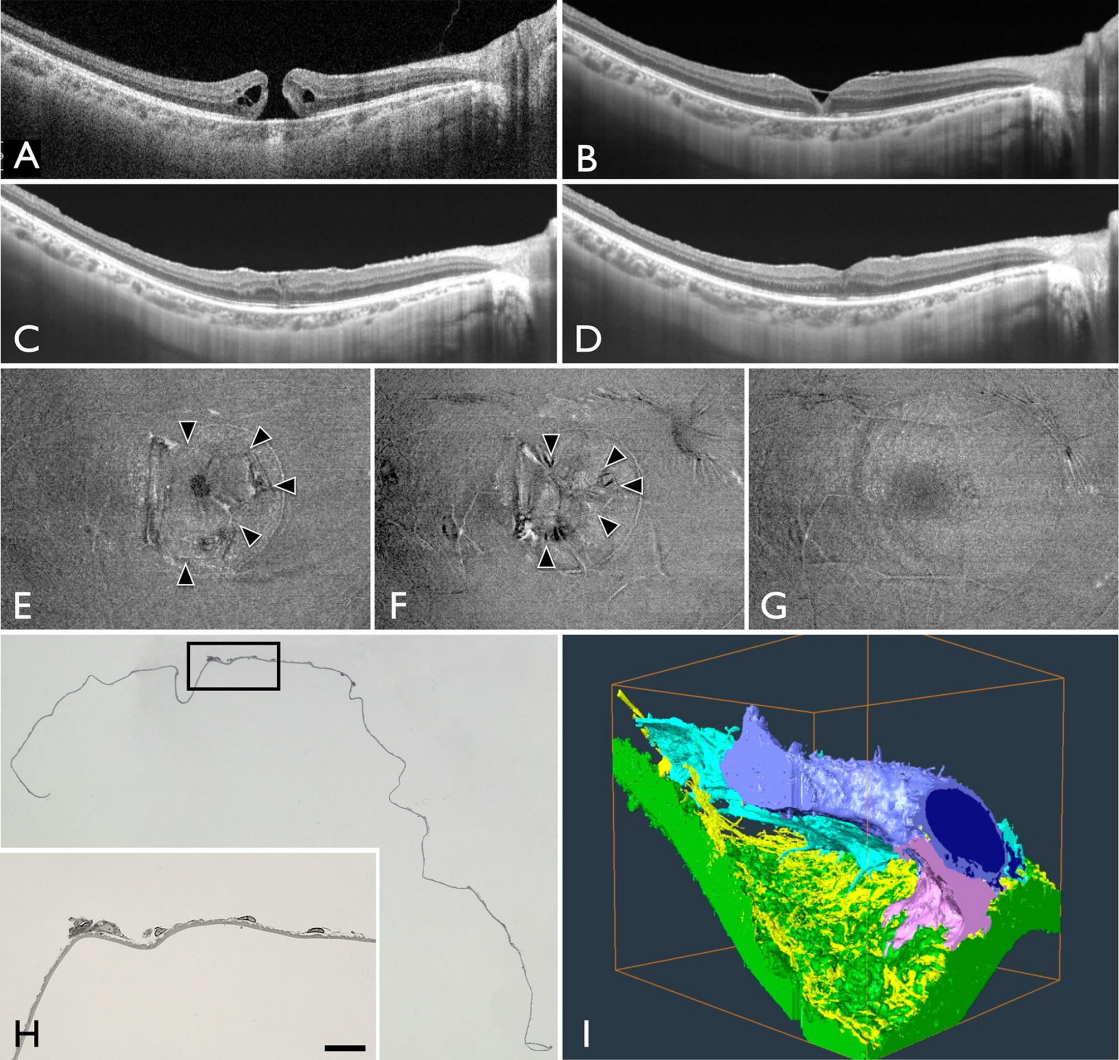
Clinical findings in a 37-year-old patient with myopic macular hole (MH) requiring reoperation. (**A**) Preoperative OCT image. The MH diameter was 290 µm and the axial length was 27.11 mm. (**B**) OCT image obtained 1 month after the surgery. The ILM flap covered the MH, and the MH was closed. (**C**) OCT image obtained 6 months after surgery. Although the patient's vision improved after the vitrectomy, the foveal depression gradually disappeared. (**D**) OCT image at 13 months after the first surgery (1 month after the second surgery). Twelve months after the first surgery, the patient underwent another vitrectomy to remove the ILM flap. One month after the surgery, the central foveal depression recovered, and BCVA improved. (**E**) OCT En Face image at 1 month postoperatively; an ILM flap with a clear border was observed covering the macula. The area of the ILM flap was 3.31 mm^2^. (**F**) OCT En Face image at 6 months postoperatively. The ILM flap had wrinkles and afferent contraction, and the area of the ILM flap was reduced to 2.59 mm^2^. (**G**) OCT En Face image at 13 months after the first surgery (1 month after the second surgery). The ILM flap was completely removed. (**H**) Light microscopy findings of the removed ILM flap. The ILM flap could be peeled off as a single sheet, and cell adhesion was observed on the surface of the ILM. The cells were found on the surface of the ILM that was originally on the retinal side, that is, the area that was newly inverted and faced the vitreous side. Bar, 10 µm. (**I**) Three-dimensional reconstructed image of the peeled ILM flap using focused ion beam-equipped scanning electron microscopy, as described previously ^36^. Multiple cells (purple, pink, and sky blue) adhered to the ILM (green) surface, and cell–cell adhesion was also observed. The fibrous component (yellow) is also seen on the surface of the ILM.

The ILM flap was clearly observed in the OCT en face image in the vitreoretinal interface region (Fig. 3). The area of the ILM flap decreased significantly from 3.25 ± 1.27 mm^2^ at 1 month to 3.13 ± 1.23 mm^2^ at 6 months (*P* = 0.024, Wilcoxon signed-rank test; Fig. 4). Two eyes with idiopathic MH and three eyes with myopic MH (a total of five eyes, 23%), showed ILM flap contraction of 5% or more. Two eyes out of the three eyes with myopic MH, had more than 20% contraction of the ILM flap. In one eye, BCVA decreased, and metamorphopsia was observed with the contraction of the ILM flap. As a result, ILM flap peeling was required (Fig. 5, Supplemental Video 1 and 2). Multiple regression analysis was performed using two factors related to ILM flap contraction: patient age and ILM flap area at 1 month postoperatively, which were selected by a stepwise method among the preoperative and early postoperative clinical findings including sex, patient age, MH size, preoperative BCVA, axial length, and ILM flap area at 1 month postoperatively. Patient age and the area of the ILM flap at 1 month postoperatively were found to be significant factors related to the contraction of the ILM flap (Table 2).

**Table 2:** Clinical factors related to ILM flap contraction.

Variables	Partial regression coefficient	Standard error	t-value	P-value	95% CI	Standardized partial regression coefficient
Intercept	73.94	6.39	11.58	< 0.001	60.57 to 87.31	
Age (years)	0.46	0.10	4.49	< 0.001	0.24 to 0.69	0.74
Size of ILM flap at 1 month (mm^2^)	− 1.97	0.89	− 2.20	0.040	− 3.84 to − 0.10	− 0.36

Multiple linear regression analysis with the contraction rate of the ILM flap as the objective variables and the selected preoperative clinical data as explanatory variables.

*ILM* internal limiting membrane.

## Discussion

In this study, we examined the surgical outcomes and postoperative morphological changes of the ILM flap in patients who underwent MH coverage with a hinged ILM flap made on the temporal side after ILM peeling. In all cases, the MH was closed and postoperative BCVA significantly improved. However, in some cases, the ILM flap contracted after the surgery.

The mechanism of MH closure by the ILM flap technique is thought to be multifactorial. The ILM flap may serve as a scaffold for Müller cell proliferation and migration during the MH closure, and may promote Müller cell activation. Furthermore, neurotrophic factors and bFGF produced by activated Müller cells and present on the surface of the ILM, may contribute to MH closure^13^. Flap closure has been reported to allow closure of large MHs that would likely remain open without this technique, and to continuously improve the integrity of the foveal structure^5^. In fact, in comparison with ILM peeling, the inverted ILM flap technique showed significantly higher closure rates than a conventional ILM peeling, in MHs greater than 400 μm^4,14–16^; in patients with myopic MH, the inverted ILM flap technique has been shown to be more effective than ILM peeling in terms of closure rate, foveal structure, and postoperative BCVA^8^. In this study, as in previous reports^15^, flap closure was observed in 6 of the 22 eyes (27%) at 1 week postoperatively, when intravitreal air had disappeared. Subsequently, these 6 eyes gradually achieved MH closure, without the need for further tamponade, and the outer retinal layers were restored. However, there has been a report of poor anatomical and visual results associated with inverted ILM flaps compared to ILM peeling, suggesting potential limitations of the ILM flap technique for repairing refractory MH^17^. Between the method of covering the MH with ILM flap and the inverted ILM flap technique that plugs the MH, the cover group had better anatomical recovery and early postoperative visual acuity^18^. In addition, the method of ILM insertion within the MH had inferior anatomical and visual results compared to the method of ILM flap covering over the MH^19^. These findings suggest that insertion of the ILM into the MH during MH surgery may interfere with the reconstruction of the outer retinal layer and impede visual recovery^20^.

Michalewska et al. reported the original method of covering the MH with an ILM around the MH, which was followed by the temporal ILM flap which covers the MH as a single sheet^10,11^, the pedicle ILM transposition flap method^21^, a large semicircular inverted ILM flap^22^, and multiple ILM flaps stacked on top of each other to resemble cabbage leaves^23^. We have devised and used a method of ILM peeling and temporal side ILM flap over the MH. The advantages of this technique are that the ERM around the MH can be completely removed, the ILM flap is large enough to cover the MH, and it can be applied to cases in which ILM peeling was previously performed. As previously reported, it is also possible to create an ILM flap above the MH^24^.

In contrast, the ILM flap may contract, as shown in this study and in previous reports^12,25^. Multiple regression analysis showed that the younger the age and the larger the ILM flap, the more it tends to contract, and the contraction of the ILM flap may lead to a decrease in postoperative visual function. Studies of epiretinal membrane (ERM) recurrence have shown that ILM peeling reduces ERM recurrence, possibly because of the presence of ERM fragments on the surface of the ILM and the fact that the ILM serves as the basis for cell proliferation and may induce cell proliferation and epiretinal membrane recurrence^26–29^. In this study, histopathological examination revealed that cells adhered to the surface of the ILM that faced the vitreous side after inversion, suggesting that cell proliferation occurred using the ILM flap as a scaffold by a mechanism similar to that of ERM recurrence, resulting in contraction of the ILM flap. As in the case of ERM recurrence, peeling of the ILM flap can be expected to improve visual function in patients with visual impairment^12^.

In this study, we presented pre- and postoperative visual acuity as an index of visual function. Similarly, metamorphopsia is one of the most important complaints after MH surgery and is thought to be related to postoperative macular deformation caused by ILM peeling^30–32^. However, it is not fully understood how the MH coverage by the ILM flap affects postoperative metamorphopsia. Although quantitative evaluation of metamorphopsia was not performed in this study, the experience of one case in which metamorphopsia was worsened by ILM flap contraction suggests that ILM flaps may be involved in postoperative metamorphopsia. The mechanism of the change in metamorphopsia due to the difference in the MH repair process after vitrectomy with the ILM flap and the progression of metamorphopsia due to the contraction of the ILM flap need to be further investigated.

The limitations of this study are the small number of cases included and the relatively short observation period of 6 months. It has been considered that morphological changes of the ILM flap could occur and should be examined over a long period of time.

In conclusion, vitrectomy using the inverted ILM flap technique yielded good anatomical and functional results. However, there are cases in which ILM flap contraction is observed during the postoperative period. Especially in younger individuals and cases with large flaps, the degree of contraction of the ILM flap tends to be large, and reoperation may be required. The ILM flap technique is a relatively new technique, and it is important to find a safe protocol that can be reliably performed, and can facilitate the maintenance of good visual function for a long period of time.

## Patients and methods

### Patients

This study included patients who underwent vitreous surgery for MH at the Hayashi Eye Hospital between August 2018 and February 2020, in which the MH was treated by vitrectomy with coverage of a single ILM flap and had a follow-up period of at least 6 months after vitrectomy. We conducted this prospective observational case series study in accordance with the Declaration of Helsinki and received approval from the Ethics Committee of the Hayashi Eye Hospital. All patients who participated in the study gave written informed consent after receiving an explanation of the nature of the study before inclusion in the study. The ILM flap technique was indicated when the hole diameter was greater than 400 µm, in the case of idiopathic MHs and myopic MHs, with an axial length of ≥ 26 mm. Eyes with diabetic retinopathy, retinal vein occlusion, a history of pars plana vitrectomy, or uveitis, were excluded.

### Surgical procedure

**Figure 6:**
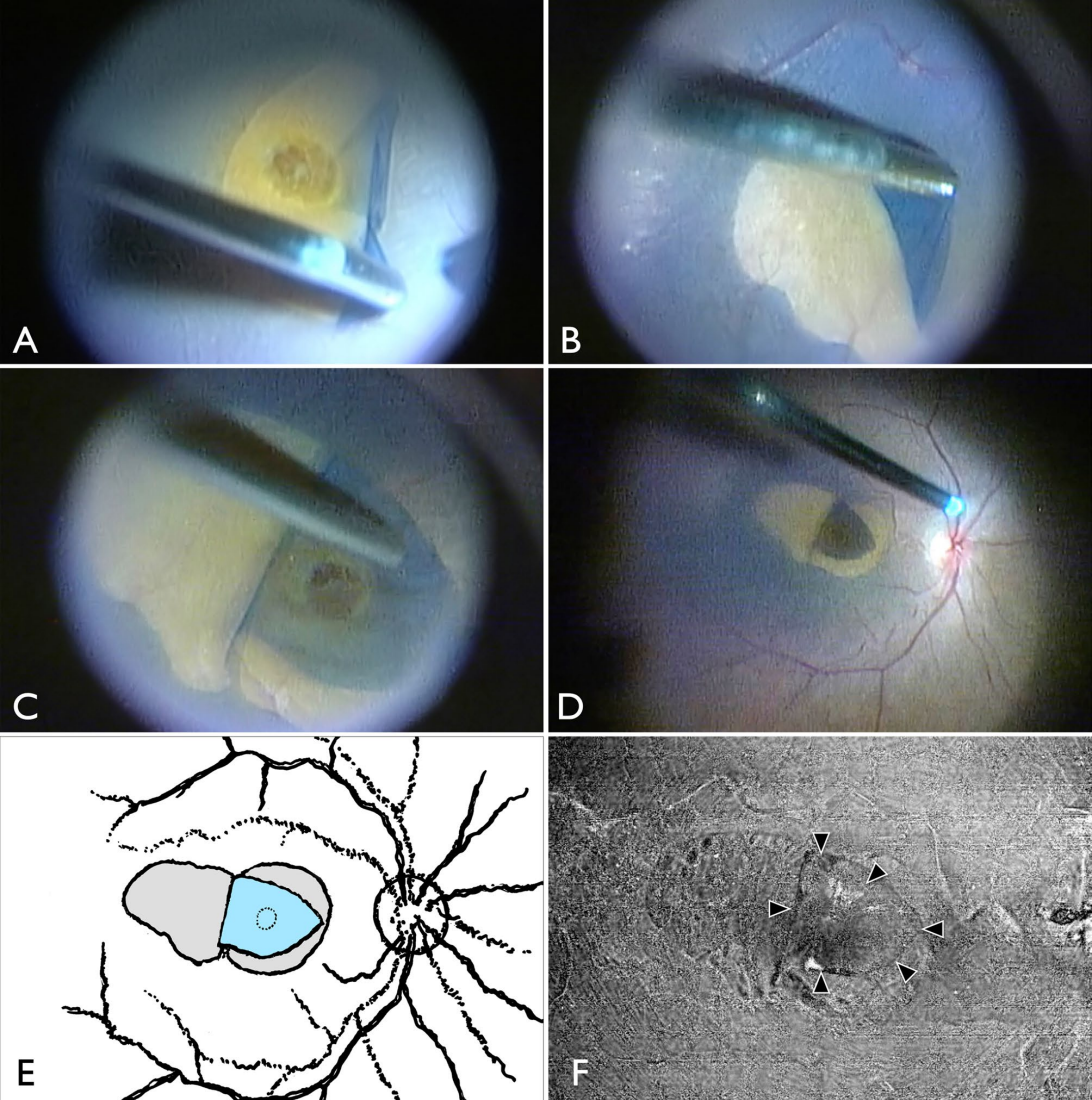
Intraoperative findings demonstrating surgical technique. (**A**) After core vitrectomy with posterior vitreous detachment, internal limiting membrane (ILM) peeling around the macular hole (MH) was performed with a radius of a one disc diameter. (**B**) An ILM flap was created at a temporal site approximately 2DD away from the ILM peeling edge, with the temporal ILM peeling edge as a hinge. (**C**) The ILM flap covered over the MH, and dispersive ophthalmic viscosurgical devices were applied to fix the ILM flap. (**D**) Fluid-air exchange was performed to fill the vitreous cavity with air. (**E**) Schematic diagram of surgical findings: ILM flap is shown in sky blue, ILM peeling area and ILM flap fabrication site are shown in gray. (**F**) OCT en face image at 6 months postoperatively. The ILM flap that was covered at the time of surgery was clearly observed.

All patients underwent surgery by a single surgeon (A.H.) following induction of local sub-Tenon’s anesthesia, as previously reported^12,33^. Phacoemulsification and aspiration (PEA) and 3-piece hydrophobic acrylic intraocular lens (IOL) implantation were performed in 15 patients with cataracts. Three patients had a clear lens that did not require cataract surgery. The remaining five patients had already undergone cataract surgery. Subsequently, we performed a 25-gauge pars plana vitrectomy using a wide-angle viewing system (Fig. 6 and Supplemental Video 3). After core vitrectomy, a posterior vitreous detachment was created in cases where posterior vitreous detachment had not occurred. After staining the macula with Brilliant Blue G solution^34^, the ILM was peeled at the size of one disc diameter around the MH (Fig. 6A). Next, an ILM flap was created on the temporal side of the macula to sufficiently cover the hole (Fig. 6B), and the flap was stretched and covered over the hole using dispersive ophthalmic viscosurgical devices (Fig. 6C). Peripheral vitrectomy was then performed while monitoring for peripheral retinal tears. Finally, fluid-air exchange and air tamponade were performed (Fig. 6D). The patient was instructed to remain in the prone position for 1–3 days postoperatively.

### Examination

Best-corrected visual acuity (BCVA) was measured using the Landolt chart at various time points including, before surgery, immediately after the disappearance of air in the eye, and at three post-operative points (1, 3, and 6 months). BCVA measurements were converted to the logarithm of the minimum angle of resolution (logMAR) for analysis. All patients underwent retinal imaging using a swept-source OCT (Plex Elite 9000; Carl Zeiss Meditec Inc., Dublin, CA, USA). An image with a signal strength of 7 or higher (up to 10) was defined as a suitable. Horizontal and vertical HD scans (HD Spotlight 1 with a measurement width of 16 mm, 0°, and 90°) were used to measure the MH diameter and to analyze the continuity of the ELM and ellipsoid zone. Cube scans (12 mm × 12 mm, 800 × 800 pixels) were used to measure the size of the ILM flap. En face structural slabs of the vitreoretinal interface were created from the cube scans by automated segmentation (133 μm above the ILM layer as the inner boundary and 33 μm below the ILM layer as the outer boundary) allowing visualization of the ILM flap boundary. The en face image obtained to measure the size of the ILM flap was saved as a TIFF file and imported with ImageJ software (https://imagej.nih.gov/ij/; provided in the public domain by the National Institutes of Health, Bethesda, MD)^35^. The boundaries of the ILM flaps were manually traced and segmented using a graphics tablet (Wacom Cintiq 16; Wacom Co., Ltd., Kazo, Saitama, Japan), and the flap area was measured using the measurement features built into ImageJ.

Before the measurement of the ILM flap area, a reconstructed image of the retinal surface was created using 3D reconstruction software to verify whether the contour of the ILM flap, obtained from the en face image of OCT, was accurate. The captured data from the cube scan were exported as a movie file, processed, and saved as a stack image using ImageJ. The data were further transferred to the 3D visualization/analysis software (FEI Amira v6.0.1 for Mac OS X; https://www.thermofisher.com/jp/en/home/electron-microscopy/products/software-em-3d-vis/amira-software.html; Thermo Fisher Scientific, Waltham, MA), and a 3D image of the macular retina was created. The obtained images showed the unevenness of the retinal surface from various directions, and the contour of the ILM was confirmed to be consistent with the contour of the ILM flap obtained from the en face image (Supplemental Video 1).

The following four issues were examined: postoperative MH status (presence or absence of MH closure and the continuity of external limiting membrane and ellipsoid zone), comparison of BCVA before and 6 months after surgery, comparison of the ILM flap area at 1 month and 6 months postoperatively, and analysis of preoperative clinical data related to changes in the ILM flap size.

### Specimen preparation and focused ion beam equipped scanning electron microscope (FIB/SEM) tomography examination

In one case that required reoperation, the ILM flap removed during surgery was immediately immersion-fixed in 2.5% glutaraldehyde solution in 0.1 M phosphate-buffered saline (PBS) for at least 2 h and then stored for further preparation. The specimen was prepared as previously described^36,37^. Briefly, after washing five times with PBS, the specimen was postfixed for 30 min in a solution containing 2% osmium tetroxide and 1.5% potassium ferrocyanide in PBS at 4 °C. The specimen was then washed with distilled water and immersed in 1% thiocarbohydrazide solution for 30 min. After washing with distilled water, the specimen was further immersed in 2% osmium tetroxide in distilled water and then washed with distilled water. Subsequently, the specimen was en bloc stained in a solution of 4% uranyl acetate solution overnight and further stained by Walton’s lead aspartate solution for 1 h for contrast enhancement. After the staining, the specimen was dehydrated in an ethanol series, followed by infiltration of epoxy resin (Epon 812; TAAB Laboratories Equipment Ltd., Berkshire, UK) mixture, and polymerized for 72 h at 60 °C. The surface of the embedded specimen was exposed using a diamond knife on an Ultracut E microtome (Leica, Wetzlar, Germany). The resin block was then placed on a standard scanning electron microscope (SEM) specimen holder with adhesives for SEM imaging.

The freshly exposed surface of the specimen was examined by backscattered electron imaging using FIB/SEM (Quanta Three-dimensional FEG, FEI, Eindhoven, The Netherlands). Serial images of the block face were acquired by repeated cycles of sample surface milling and imaging using Slice and View G2 software (FEI). The milling pitch was set to 50 nm/step and 800 cycles. The resultant image stack was processed using the ImageJ (National Institutes of Health, Bethesda, MD) and FEI Amira v6.0.1 software (Thermo Fisher Scientific, Waltham, MA). To observe the three-dimensional structures of the specimen, the ILM and the membrane components of the cells were semi-automatically segmented. Subsequently, the outlined ILM and cells were then visualized and displayed.

### Statistical analysis

In comparing the clinical characteristics of patients with idiopathic MH and myopic MH, we used an unpaired *t* test for patient age, MH size, preoperative BCVA, and axial length, and a Fisher's exact probability test for sex. Comparison of pre-and postoperative BCVA was performed using the paired *t* test, and the ILM flap area was compared using en face images at the vitreoretinal interface and tested using the Wilcoxon signed-rank test. The Spearman’s rank correlation coefficient was calculated for the correlation between postoperative BCVA after 6 months and preoperative factors including patients age, sex, MH size, preoperative BCVA and axial length, and the size of the ILM flap at 1 month. The factors related to ILM flap contraction were analyzed by multiple linear regression analysis with the objective variables being the contraction rate of the ILM flap and the preoperative clinical data selected by the stepwise method as explanatory variables. All statistical analyses were performed using JMP^®^ Pro 15 statistical software (SAS Institute, Inc., Cary, NC, USA) or Prism 7 for Mac OS X (version 7.0b, GraphPad Software, Inc., San Diego, CA, USA), and statistical significance was set at *P* < 0.05.

## Supplementary Information

Supplementary Video 1.

Supplementary Video 2.

Supplementary Video 3.

Supplementary Legends.

## Data Availability

The datasets used and/or analyzed during the current study are available from the corresponding author on reasonable request.
