# Supplementary material for: Contractility of temporal inverted internal limiting membrane flap after vitrectomy for macular hole
Source: Sci Rep. 2021 Oct 8;11:20035. doi: 10.1038/s41598-021-99509-0 (PMC8501065; doi:10.1038/s41598-021-99509-0)
Supplement: Supplementary file 4 — Supplementary Legends. [file 41598_2021_99509_MOESM4_ESM.pdf]

Supplementary video legends

Supplemental video 1

OCT En Face images and corresponding 3-dimentional reconstructed images at 1 and 6 months postoperatively. The contours of the internal limiting membrane (ILM) obtained from the en face image were consistent with the contours of the ILM flap images created by 3D reconstruction software from the stacked images of the cube scan.

Supplemental video 2

3D reconstructed image of the peeled internal limiting membrane (ILM) flap

The upper part of the image obtained by focused ion beam-equipped scanning electron microscopy shows the original retinal side of the ILM, which has been inverted after surgery and is facing the vitreous side; multiple cells (purple, pink, and light blue) are attached to the surface of the ILM (green), and cell-cell adhesion is also observed; the fibrous component (yellow) is also seen on the surface of the ILM.

Supplemental video 3

Intraoperative findings demonstrating surgical technique

After staining the macula with Brilliant Blue G solution, the internal limiting membrane (ILM) peeling around the macular hole (MH) was performed. Then, an ILM flap was created on the temporal side of the macula and covered over the hole using dispersive ophthalmic viscosurgical devices. Finally, fluid-air exchange and air tamponade were performed.
